# Boosting with Multiple Doses of mRNA Vaccine after Priming with Two Doses of Protein Subunit Vaccine MVC-COV1901 Elicited Robust Humoral and Cellular Immune Responses against Emerging SARS-CoV-2 Variants

**DOI:** 10.1128/spectrum.00609-22

**Published:** 2022-08-25

**Authors:** Chun-Hsiang Chiu, Yu-Hsiu Chang, Chi-Wei Tao, Feng-Yee Chang, Kuo-Chou Chiu, Tien-Wei Chang, Li-Chen Yen

**Affiliations:** a Division of Infectious Diseases and Tropical Medicine, Department of Internal Medicine, Tri-Service General Hospital, National Defense Medical Center, Taipei, Taiwan; b Institute of Preventive Medicine, National Defense Medical Center, Taipei, Taiwan; c Division of Chest Medicine, Department of Internal Medicine, Cheng-Hsin General Hospital, Taipei, Taiwan; d Department of Family Dentistry, Tri-Service General Hospital, National Defense Medical Center, Taipei, Taiwan; e School of Dentistry, National Defense Medical Center, Taipei, Taiwan; f Department of Microbiology and Immunology, National Defense Medical Center, Taipei, Taiwan; University of Georgia

**Keywords:** SARS-CoV-2, viral variants, heterologous vaccination, protein subunit vaccine, neutralizing antibody, cellular immune response, emerging variants, neutralizing antibodies

## Abstract

Confronted with severe acute respiratory syndrome coronavirus 2 (SARS-CoV-2) variants, such as Delta and Omicron, with high infectivity and immune evasion capacity, vaccination remains the most effective tool to prevent infection and severe illness. However, heterologous vaccination of mRNA vaccines primed with protein subunit vaccines had not been evaluated before the current study. Since subunit vaccine MVC-COV1901 (MVC) has been granted emergency use authorization in Taiwan, in this study, we explored the humoral and cellular immune responses to additional third (2× MVC/Mod) and fourth (2× MVC/2× Mod) doses of mRNA-1273 (Mod) after priming with two doses of subunit vaccine MVC against the emerging variants. We found a 12.3- to 16.1-fold increase in antibodies targeting the receptor binding domain (RBD) of the Delta variant with 2× MVC/Mod compared to two doses of MVC (2× MVC) or AZD1222 (2× AZ) regimens and a 26- to 32.2-fold improvement in neutralizing potency against the Omicron variant (BA.1). Besides, the numbers of gamma interferon (IFN-γ)-secreting T cells induced by 2× MVC/Mod were also elevated 3.5-fold and 3.7- to 4.3-fold for the wild type and Delta variant. However, boosting with a fourth dose of Mod (2× MVC/2× Mod) after the 2× MVC/Mod regimen failed to significantly improve the immune responses. Moreover, all vaccination schedules showed reduced neutralizing activity against the Omicron variant. Collectively, our results suggested that the third or fourth dose booster vaccination with mRNA vaccine after priming with two doses of protein subunit vaccine could elicit stronger humoral and cellular immune responses. These findings could provide a future global heterologous boosting strategy against COVID-19.

**IMPORTANCE** Vaccination is the most important strategy to combat the COVID-19 outbreak; however, it remains to be determined whether heterologous prime-boost regimens could induce equal or even stronger immune responses against SARS-CoV-2. Here, we showed that boosting the additional doses of mRNA-1273 (Mod) priming with two doses of MVC-COV1901 (MVC) (2× MVC/Mod) improved humoral and cellular immunity compared to two doses of AZD1222 (2× AZ) or MVC (2× MVC) against SARS-CoV-2 variants. However, the Omicron variant showed strong immune evasion ability for all vaccination schedules. Our findings provided evidence supporting that heterologous vaccination by boosting with mRNA vaccine after priming with two doses of protein subunit vaccine could strongly promote humoral and cellular immune responses against the emerging SARS-CoV-2 variants.

## INTRODUCTION

The outbreak of coronavirus disease 2019 (COVID-19) caused by severe acute respiratory syndrome coronavirus 2 (SARS-CoV-2) threatens public health. Vaccination is an important countermeasure to combat pandemics, and multiple vaccines have been approved for use, including the mRNA vaccines Spikevax (mRNA-1273; Moderna [here referred to as Mod]) and Comirnaty (BNT162b2; Pfizer-BioNTech [here referred to as BNT]), the viral vector-based vaccine Vaxzevria (ChAdOx1-nCoV-19; AstraZeneca [here referred to as AZ]), and the protein subunit vaccine Covovax (NVX-CoV2373; Novavax [here referred to as NVX]), all of which were authorized for homologous or heterologous prime-boost schedules elsewhere (1, 2).

AZ is the first COVID-19 vaccine granted emergency use authorization (EUA) in Taiwan, and recent studies have suggested that boosting AZ-primed individuals with Mod (3) or BNT (4–6) is more reactogenic and induces superior humoral and cellular immune responses than homologous vaccination. In addition, an Israeli study showed that receiving a third dose of BNT compared with receiving only two doses of BNT at least 5 months prior was more effective at preventing severe illness and death (7). Another study showed that kidney transplant recipients who received a third dose of BNT had higher neutralizing antibody titers and T cell responses than those who received only two doses of BNT (8).

The MVC-COV1901 COVID-19 vaccine (here referred to as MVC) is a protein subunit vaccine based on the recombinant S-2P antigen adjuvanted with CpG 1018 supplied by Dynavax and aluminum hydroxide. The S-2P antigen is a trimeric and prefusion-stable recombinant spike protein developed by the U.S. National Institutes of Health (NIH). CpG 1018 was shown to enhance immunogenicity and induce a Th1-skewed immune response (9). MVC has been granted emergency use authorization (EUA) in Taiwan, with vaccinations commencing in August 2021. Phase 1 (10) and phase 2 (11) trials have shown that MVC was well tolerated and elicited robust humoral and cellular immune responses.

Due to the continued emergence of new variants of SARS-CoV-2, heterologous vaccination with certain combinations may improve the immune responses compared to homologous vaccination. Although a few third-dose homologous and heterologous booster studies have been conducted, immunogenicity of heterologous vaccination of mRNA vaccine primed with protein subunit vaccines had not been evaluated before the current study. Thus, in this study, we aimed to investigate the effect of third or fourth booster shots of Mod on humoral and cellular immune responses in individuals primed with two doses of MVC against emerging SARS-CoV-2 variants.

## RESULTS

### Clinical characteristics of the participants enrolled in this study.

Blood samples were obtained from 29 vaccinated individuals during August 2021 to December 2021. Among the members of group A (who received two doses of AZ [2× AZ]), 8 (53.3%) were male and 7 (46.7%) were female, with a median age (interquartile range [IQR], 25 to 75 years) of 40 years (range, 28 to 44 years), while among the members of group B (who received two doses of MVC [2× MVC]), 6 (42.9%) were male and 8 (57.1%) were female, with a median age (IQR, 25 to 75 years) of 44.5 years (range, 32 to 54.25 years). Groups C and D were participants derived from group B who were administered an additional third (2× MVC/Mod) or fourth (2× MVC/2× Mod) dose of Mod. The demographic details of the participants in this study were balanced among the study groups and are summarized in Table 1.

**TABLE 1:** Demographic and clinical characteristics of vaccinated individuals enrolled in this study

Gender	Age (yr)	Underlying disease	Standard prime doses	Booster doses	Interval(s) between 2nd/3rd/4th doses
M	61	Hypertension	2× AZ		72
F	44		2× AZ		81
M	40		2× AZ		76
M	28		2× AZ		73
M	40		2× AZ		78
F	39		2× AZ		74
F	32		2× AZ		77
M	42		2× AZ		82
M	23		2× AZ		70
M	23		2× AZ		71
F	23		2× AZ		75
M	41		2× AZ		75
F	32		2× AZ		73
F	50		2× AZ		74
F	45	Hypertension	2× AZ		79
M	25		2× MVC	2× Mod	28/34/29
F	29		2× MVC	2× Mod	30/32/31
M	62	Hypertension	2× MVC	2× Mod	32/29/28
M	55	Hypertension	2× MVC	2× Mod	29/31/30
F	32		2× MVC	2× Mod	28/33/32
M	51		2× MVC	2× Mod	31/30/30
M	43		2× MVC	2× Mod	32/28/33
F	56	Hyperlipidemia	2× MVC	2× Mod	30/32/31
M	54		2× MVC	2× Mod	33/29/28
F	51		2× MVC	2× Mod	28/28/31
F	45		2× MVC	1× Mod	30/31
F	34		2× MVC		31
F	32		2× MVC		29
F	44		2× MVC		34

### Measurement of Alpha and Delta variant anti-RBD IgG titers under different vaccination schedules.

**FIG 1:**
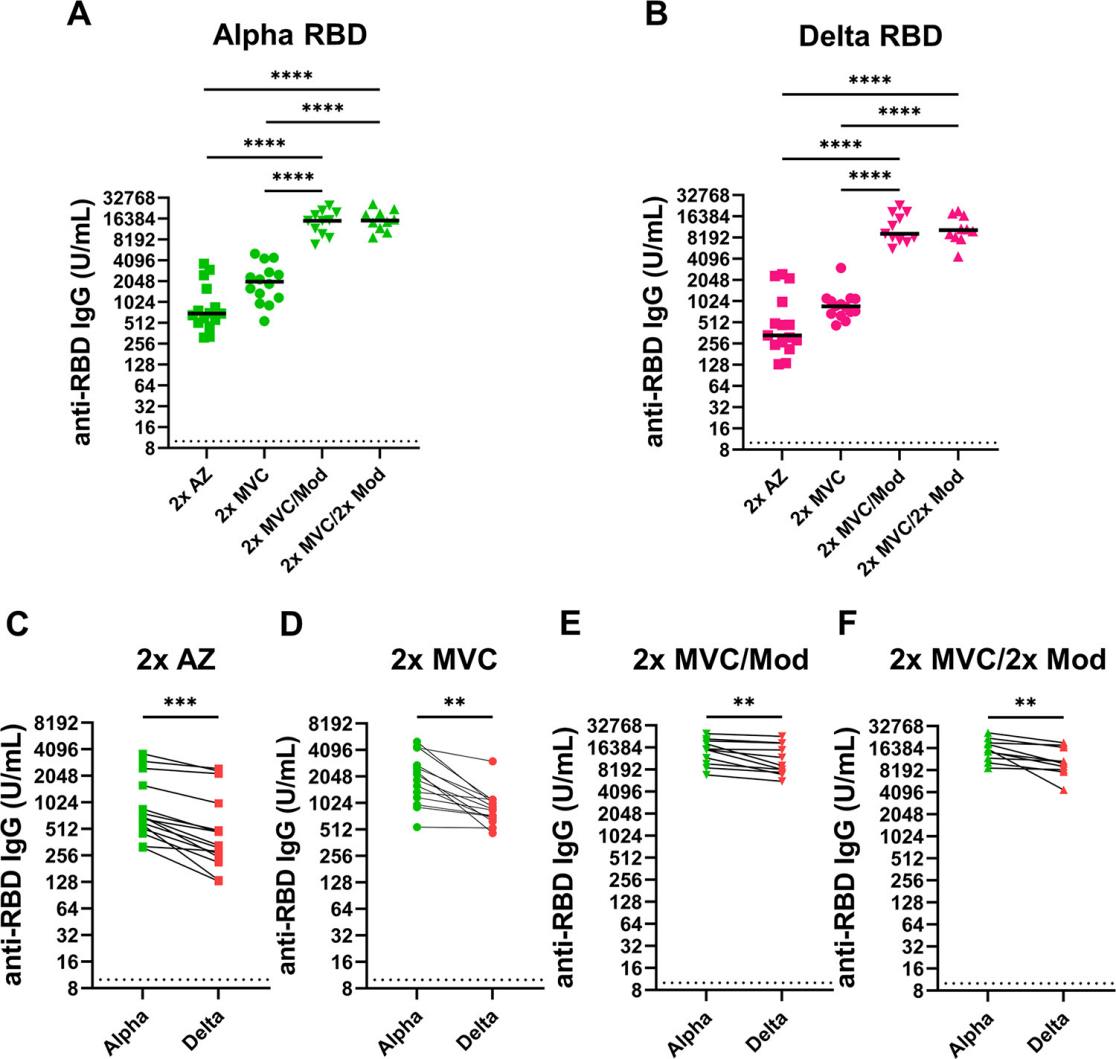
Measurement of Alpha and Delta variant anti-RBD IgG titers under different immunization schedules. (A and B) Results of ELISAs measuring serum reactivity to anti-RBD IgG for the (A) Alpha variant and (B) Delta variant. (C to F) Comparison of the differences between Alpha and Delta variant anti-RBD IgG titers for the same vaccination schedules. (C) 2× AZ; (D) 2× MVC; (E) 2× MVC/Mod; (F) 2× MVC/2× Mod. Duplicates were performed for each tested sample. Statistical significance was calculated among experiments by one-way ANOVA with Tukey's multiple-comparison test or two-tailed Student's *t* test. The dotted line represents the cutoff value for each assay. Asterisks indicate statistical significance: **, *P* < 0.01; ***, *P* < 0.001; ****, *P* < 0.0001.

Since AZ was the first COVID-19 vaccine to be approved in Taiwan, it was chosen as the comparator vaccine with locally developed MVC (12). We first measured the RNA-binding domain (RBD)-specific IgG titers of individuals with different vaccination schedules against different SARS-CoV-2 variants using enzyme-linked immunosorbent assay (ELISA) (Fig. 1). The results showed no significant differences between individuals administered two doses of AZ (2× AZ) or two doses of MVC (2× MVC) for the Alpha (adjusted *P* value [*p*.adj] = 0.8415) and Delta (*p*.adj = 0.9980) variants. However, in individuals boosted with a single dose of Mod after priming with two doses of MVC (here referred to as 2× MVC/Mod), the median (IQR, 25 to 75) anti-RBD IgG titers against the Alpha and Delta variants were 15,275.68 (range, 10763.54 to 19,207.94) and 9,250.81 (range, 7,826.68 to 16,938.11), which were 13.4- and 16.1-fold higher than those with 2× AZ, respectively, or 6.7- and 12.3-fold higher than those with 2× MVC, respectively (*p*.adj < 0.0001) (Fig. 1A and B). There were no significant differences in anti-RBD IgG titers for the Alpha (*p*.adj = 0.9317) or Delta (*p*.adj = 0.9779) variant in individuals who were boosted with two additional doses of Mod after priming with two doses of MVC (here referred to as 2× MVC/2× Mod) compared to those with 2× MVC/Mod. In the further comparison of the sensitivities of anti-RBD IgG for different variants for individuals with the same vaccination schedule, 2× AZ, 2× MVC, 2× MVC/Mod, and 2× MVC/2× Mod showed 1.5-fold (*P* = 0.0002), 2.3-fold (*P* = 0.0014), 1.3-fold (*P* = 0.0037), and 1.4-fold (*P* = 0.0024) declines in anti-RBD IgG titers, respectively, against the Delta variant (Fig. 1C to F). These results suggested that boosting with an additional dose of Mod after priming with two doses of MVC significantly increased anti-RBD IgG titers for Alpha and Delta variants; however, when faced with the Delta variant, which has accumulated a vast number of mutations in the RBD region, the levels of antibodies targeting the RBD are significantly reduced, regardless of the vaccination schedules.

### Comparison of the neutralizing efficacies of different vaccination schedules against SARS-CoV-2 pseudovirus and infectious virus variants.

**FIG 2:**
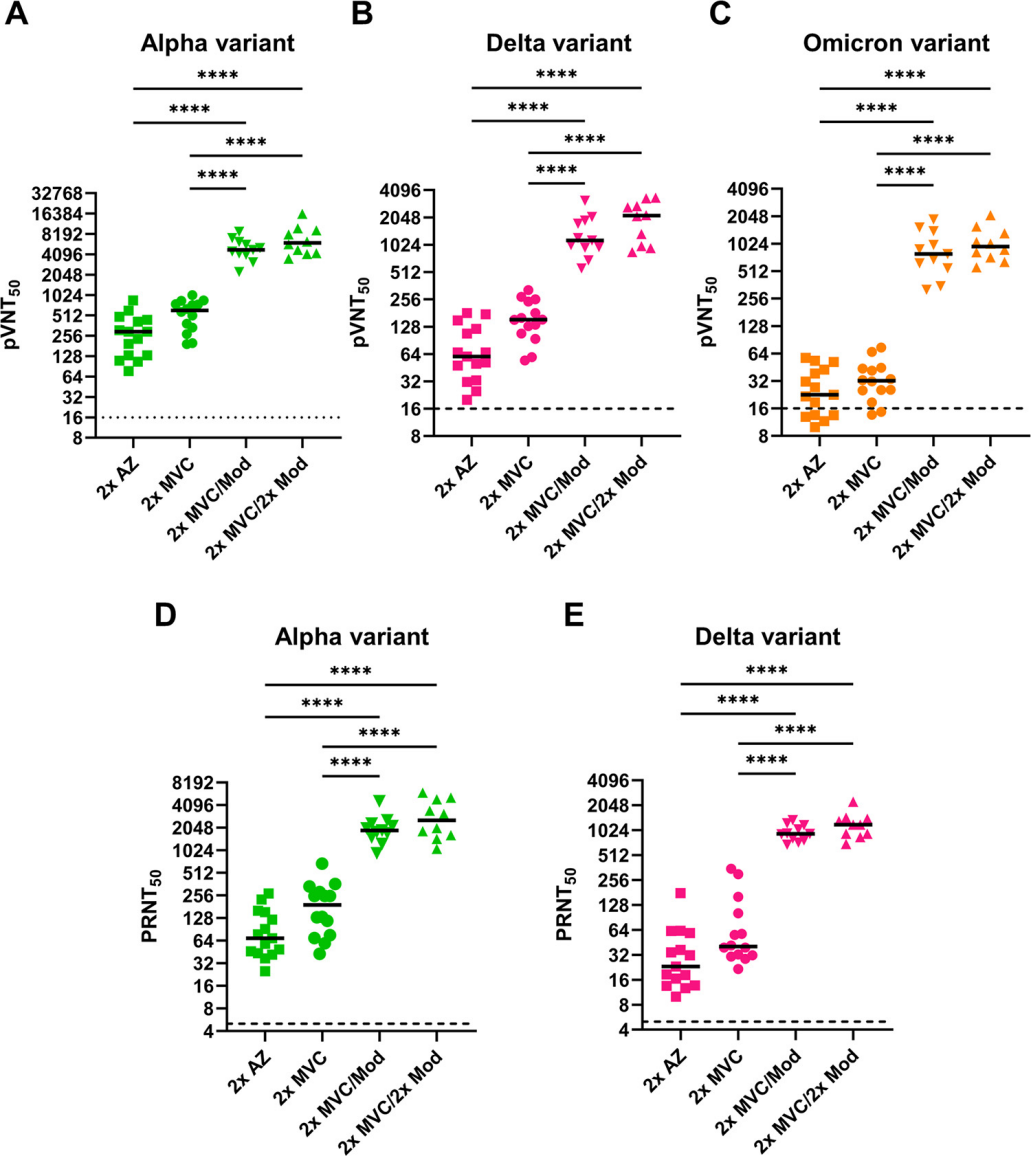
Comparison of the neutralizing efficacies of different immunization schedules against SARS-CoV-2 pseudovirus and infectious virus variants. (A to C) Results of pseudovirus neutralization (pVNT_50_) for the (A) Alpha variant, (B) Delta variant, and (C) Omicron variant; (D and E) results of infectious SARS-CoV-2 neutralization (PRNT_50_) for the (D) Alpha variant and (E) Delta variant. Duplicates were performed for each tested serum. Statistical significance was calculated among experiments by one-way ANOVA with Tukey's multiple-comparison test. The dotted line represents the cutoff value for each assay. Asterisks indicate statistical significance: ****, *P* < 0.0001.

Next, we further measured neutralizing titers against the emerging variants using pseudovirus and infectious virus (Fig. 2). There was no significant difference in the abilities of 2× AZ and 2× MVC to induce the neutralizing responses against the Alpha (*p*.adj = 0.9825), Delta (*p*.adj = 0.9751), and Omicron (*p*.adj > 0.9999) variant pseudoviruses. In comparison, the median of 50% pseudovirus neutralization titer (pVNT_50_) values (IQR, 25 to 75) with 2× MVC/Mod for the Alpha, Delta, and Omicron variants were 4,757.36 (range, 4,116.28 to 5,958.05), 1,142.49 (range, 959.80 to 1,822.13), and 789.35 (range, 587.39 to 1,199.37), respectively, which were 16.3-, 17.7-, and 32.2-fold higher than those with 2× AZ and 8.8-, 8.4-, and 26.0-fold higher than those with 2× MVC, respectively (Fig. 2A to C). The neutralizing potency of 2× MVC/2× Mod was further enhanced compared to that of 2× MVC/Mod, although there was no statistically significant difference for the Alpha (*p*.adj = 0.0678), Delta (*p*.adj = 0.0525), or Omicron (*p*.adj = 0.6666) variant. Compared to that against the Alpha variant, the levels of neutralization effectiveness of 2× AZ, 2× MVC, 2× MVC/Mod, and 2× MVC/2× Mod against the Delta variant decreased by 3.9 times (*p*.adj = 0.0105), 3.5 times (*p*.adj = 0.0245), 3.6 times (*p*.adj = 0.0570), and 3.5 times (*p*.adj = 0.0760), respectively, or 11.0 times, 16.4 times, 5.6 times, and 6.7 times (*p*.adj < 0.0001), respectively, against the Omicron variant (see Fig. S1A to D in the supplemental material). In addition, 2× MVC/Mod and 2× MVC/2× Mod also showed the highest neutralization efficacy against infectious SARS-CoV-2 for both the Alpha and Delta variants (Fig. 2D and E). The pVNT_50_ and 50% plaque reduction/neutralization (PRNT_50_) values for each participant are provided in Table S1.

Correlation analysis between anti-RBD IgG titers and pVNT_50_ or PRNT_50_ values also showed that the neutralizing titers measured with either pseudovirus or infectious virus were highly correlated with the titers of the corresponding RBD-specific antibodies against the viral variants, indicating that the neutralizing efficacy was mainly contributed by the antibodies targeting the RBD (13, 14) (Fig. S2A to D).

These data suggested that heterologous vaccination of mRNA vaccine primed with two doses of the protein subunit vaccine could significantly enhance the neutralizing abilities against the Alpha, Delta, and Omicron variants. Besides, despite all vaccination schedules being significantly less effective at neutralizing the Omicron variant, boosting with a single dose of Mod after priming with two doses of MVC could still improve the neutralizing capacity against it.

### Cellular immune response against SARS-CoV-2 with different vaccination schedules.

**FIG 3:**
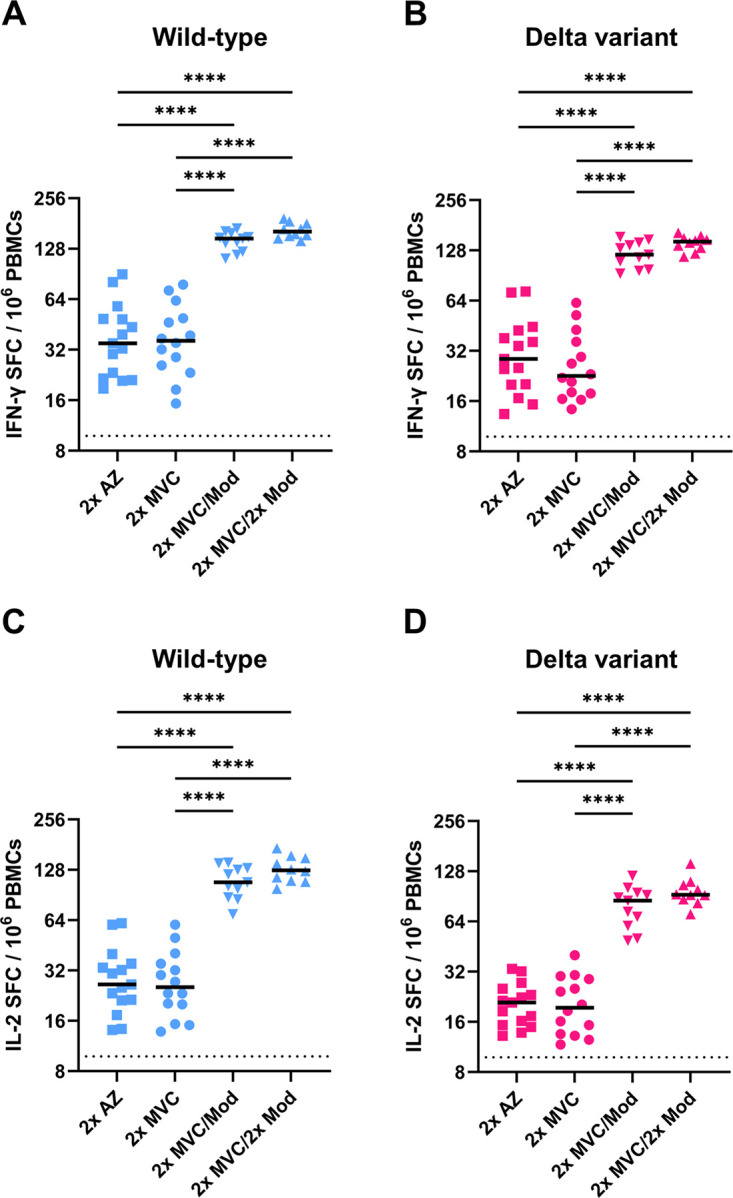
Cellular immune response against SARS-CoV-2 with different vaccination schedules. (A and B) IFN-γ ELISpot assay for different vaccination schedules, with PBMCs stimulated with the (A) wild-type or (B) Delta variant peptide pool; (C and D) IL-2 ELISpot assay for different vaccination schedules, with PBMCs stimulated with the (C) wild-type or (D) Delta variant peptide pool. SFC, spot-forming cells. Duplicates were performed for each group of tested PBMCs. Statistical significance was calculated among experiments by one-way ANOVA with Tukey's multiple-comparison test. The dotted line represents the cutoff value for each assay. Asterisks indicate statistical significance: ****, *P* < 0.0001.

Previous studies have suggested that T cell responses may have a protective role in the presence of suboptimal humoral immune responses (15). Thus, we used an enzyme-linked immunosorbent spot (ELISpot) assay to assess the cellular immune response of vaccinated individuals against SARS-CoV-2 (Fig. 3). The results showed no significant difference in gamma interferon (IFN-γ)-secreting T cell responses between 2× AZ and 2× MVC, either for the wild type (*p*.adj = 0.9998) or the Delta variant (*p*.adj = 0.8628), whereas the medians (IQR, 25 to 75 spot-forming cells [SFCs]/10^6^ peripheral blood mononuclear cells [PBMCs]) of IFN-γ-secreting T cells were 147.3 SFCs/10^6^ PBMCs (range, 130.8 to 154.7 SFCs/10^6^ PBMCs) for the wild-type and 120.6 SFCs/10^6^ PBMCs (range, 104.3 to 140.2 SFCs/10^6^ PBMCs) for the Delta variant with 2× MVC/Mod, which were significantly higher than those with 2× AZ (*p*.adj < 0.0001) and 2× MVC (*p*.adj < 0.0001), while 2× MVC/2× Mod resulted in the medians (IQR, 25 to 75 SFCs/10^6^ PBMCs) IFN-γ-secreting T cells for the wild type and the Delta variant further increasing to 161.8 (range, 152.8 to 177.2 SFCs/10^6^ PBMCs) and 144.3 (range, 133.4 to 150.5 SFCs/10^6^ PBMCs) SFCs/10^6^ PBMCs, although these levels were not significantly different from those with 2× MVC/Mod (Fig. 3A and B). We also measured the interleukin-2 (IL-2)-secreting T cell response to the wild type and Delta variant, and the results were similar to those of IFN-γ-secreting T cells, with 2× MVC/Mod and 2× MVC/2× Mod producing significantly better responses than 2× MVC and 2× AZ (Fig. 3C and D). These results suggested that administration of an additional dose of mRNA vaccine after priming with two doses of the subunit vaccine can significantly enhance the cellular immune response for both the wild type and the Delta variant.

## DISCUSSION

Heterologous vaccination against SARS-CoV-2 is important to provide alternative vaccination strategies and program flexibility. In this study, we found that boosting with a third or fourth dose of Mod after priming with two doses of the subunit vaccine MVC produced a stronger humoral immune response against the Alpha, Delta, and Omicron variants, and PBMCs stimulated with wild-type or Delta variant spike peptide pools also showed a substantial enhancement of T cell responses. Although the fourth dose of Mod could further improve the humoral and cellular immune responses toward SARS-CoV-2 variants compared to those with 2× MVC/Mod, the difference was not statistically significant. In addition, regardless of the vaccine schedules, there was a significant decrease in the neutralizing efficacy against the Omicron variant, ranging from a 5.6- to 16.4-fold reduction (see Fig. S1A to D in the supplemental material).

Previous studies have shown that heterologous AZ-BNT vaccination produces stronger humoral and cellular immune responses (4, 16) and is more effective in protection than homologous AZ/AZ (17); however, individuals boosted with subunit vaccine NVX after BNT prime did not show the superiority of spike-specific antibodies compared to homologous BNT (4), and the administration of two doses of AZ followed by an additional dose of AZ did not enhance the cellular immune responses (18, 19). Another study revealed that the booster shot not only enhanced the neutralization ability against wild-type SARS-CoV-2, but also enhanced the broadness and cross-reactivity of the humoral immune response toward variants of concern (VOCs), such as the Omicron variant (20). In addition, our results demonstrated that after priming with two doses of subunit vaccine MVC, the subsequent boosting single dose of mRNA vaccine Mod elicits superior humoral and cellular immune responses, even in the face of emerging variants with strong immune evasion capabilities.

Although compared to 2× MVC/Mod, 2× MVC/2× Mod slightly improved the humoral and cellular immune responses, the improvement was not significant, probably because the immune response was still at a plateau and the dosing interval from the third to the fourth dose was too close. Further studies are needed to investigate the efficacy of the fourth dose of vaccine and its effect on the immune response.

T cells recognize highly conserved regions in viral proteins and are less susceptible to immune evasion via mutation, even when they encounter variants of concern that have the ability to escape neutralizing antibodies (21, 22). In our results, although all vaccination schedules resulted in slightly less sensitive to the Delta variant than the wild type regarding IFN-γ- and IL-2-secreting T cells (Fig. 3A and C versus Fig. 3B and D), 2× MVC/Mod and 2× MVC/2× Mod still produced a significant increase in the overall T cell response compared to that with 2× AZ and 2× MVC, indicating that these variants can still be recognized by T cells and that these schedules can prevent worse outcomes in the face of emerging variants that are resistant to humoral immune responses.

Recent studies have shown that the neutralizing efficacy of existing COVID-19 vaccines against the Delta and Omicron variants is significantly reduced, and breakthrough infections have been reported (23–25). Our data also show that despite the stronger humoral immune response induced by heterologous prime-boost vaccination, this vaccination schedule still produced lower neutralizing ability against the Omicron variant (Fig. S1A to D), suggesting that current vaccines designed with the spike protein sequence of the wild-type SARS-CoV-2 are indeed less effective at combating variants with immune evasion capabilities. Hence, the development of next-generation COVID-19 vaccines against circulating variants is urgently needed.

This study has a few limitations. There are small sample sets with convenience sampling in this observational study, and the age range of the study cohort (25 to 62 years) limits the generalization of the immunogenicity results to children, older populations, and people who are not of Asian descent. In addition, different heterologous vaccination dose intervals and orders of priority may have distinct impacts on the efficacy of the vaccine; whether priming with mRNA vaccines followed by subunit vaccines may have a divergent effect on the immune response remains an issue to be explored. Furthermore, due to the short follow-up period, we were unable to investigate the dynamic humoral and cellular immune response changes induced by the heterologous vaccine schedules. Moreover, recent Omicron subvariants BA.4 and BA.5 have gradually become dominant strains, and current studies have shown that they have a stronger immune evasion ability than BA.1 and BA.2 (26–28); the neutralizing effectiveness of heterologous vaccination against these Omicron subvariants needs to be further evaluated.

In conclusion, after two doses of the protein subunit vaccine MVC as the “priming” shots, a third heterologous booster of mRNA vaccine was highly immunogenic in healthy adults, which significantly recalled and enhanced immune responses against SARS-CoV-2 and its variants. Our findings provide important evidence for establishing a future global heterologous boosting strategy for COVID-19.

## MATERIALS AND METHODS

### Cell lines and viruses.

BHK-21 cells, a baby hamster kidney cell line (ATCC CCL-10), were cultured in RPMI 1640 medium containing 5% fetal bovine serum (FBS) (Gibco), and HEK293 cells, a human embryonic kidney cell line (ATCC CRL-1573), were grown in Dulbecco’s modified Eagle’s medium (DMEM) containing 10% FBS. Vero E6 cells (an African green monkey kidney cell line; ATCC CRL-1586) were cultivated in high-glucose DMEM supplemented with 10% FBS and an antibiotic and antimycotic (Gibco) in a humidified atmosphere of 37°C and 5% CO_2_. SARS-CoV-2 B.1.1.7 (hCoV-19/Taiwan/792/2020; GISAID accession ID EPI_ISL_411927, Alpha variant) and B.1.617.2 (hCoV-19/Taiwan/1144/2021; GISAID accession ID EPI_ISL_5854263, Delta variant) were provided by the Taiwan Centers for Disease Control, Ministry of Health and Welfare, and propagated using Vero E6 cells supplemented with 2% FBS. Third-passage virus was used for all of the studies described here. Viral stocks were free of contamination, and viral titers were determined by plaque assay, followed by storage of aliquots at −80°C until further use in experiments.

### Participants.

In this observational study, a convenience sample of 29 adult participants (ages of 25 to 62 years) with good physical health (mild-to-moderate well-controlled comorbidities were permitted) and with no history of laboratory-confirmed SARS-CoV-2 infection who had received at least 2 standard prime doses of either AZ or MVC were enrolled at a COVID-19 vaccination site that was set up in the Tri-Service General Hospital in Taiwan. The median (IQR, 25 to 75 days) duration between the two doses of AZ was 75 days (range, 73 to 78 days), and the median (IQR, 25 to 75 days) duration between the two doses of MVC was 30 days (range, 28.75 to 32 days). According to the booster COVID-19 vaccines they had received, they were then divided into 4 subgroups: A (*n* = 15), B (*n* = 14), C (*n* = 11), and D (*n* = 10). Participants in groups C and D were followed up from group B. Participants in group A did not receive the booster vaccine and had received two standard prime doses of AZ (2× AZ) (0.5 mL/dose contained 5 × 10^10^ viral particles via intramuscular injection). Participants in group B also did not receive the booster vaccine and had received two standard prime doses of MVC (2× MVC) (0.5 mL/dose contained 15 μg of CHO cell-produced S-2P protein, adjuvanted with 750 μg CpG 1018 and 375 μg aluminum hydroxide via intramuscular injection). The participants followed up in group C received a single dose of Mod (100 μg administered at 0.5 mL via intramuscular injection) after the second shot of the prime vaccine, MVC (2× MVC/Mod), and the median (IQR, 25 to 75 days) duration was 31 days (range, 29 to 32 days). Also, the participants followed up in group D received the fourth booster dose of Mod after the third shot of the Mod (2× MVC/2× Mod), and the median (IQR, 25 to 75 days) duration was 30.5 days (range, 28.75 to 31.25 days). Blood samples were obtained 14 days after the second, third, and fourth vaccine doses. All adverse events were mild or moderate and mostly disappeared 3 days after the booster vaccination. The study was approved by the Institutional Review Board of Tri-Service General Hospital, and informed consent was obtained from the included participants.

### Spike plasmid cloning and SARS-CoV-2 pseudovirus production.

Construction of pseudovirus that carrying the spike protein of SARS-CoV-2 was performed as previously described (29). In brief, 60 μL Lipofectamine 3000 transfection reagents (Thermo Fisher) was mixed with 500 μL serum-free DMEM, held at room temperature for 5 min, and then mixed with the following plasmids that were diluted in 500 μL serum-free DMEM for another 20 min: pLAS3w-FLuc-Ppuro (9.5 μg) and pCMV-Δ8.91 (Gag-Pol provider, 6.5 μg), the spike plasmids (4.5 μg) pcDNA3.3_CoV2_B.1.1.7 (Addgene no. 170451) and pcDNA3.3-SARS2-B.1.617.2 (Addgene no. 172320), and the SARS-CoV-2 Omicron Strain S gene Human codon_pcDNA3.1(+) plasmid (B.1.1.529/BA.1) (GenScript no. MC_0101274). This DNA-Lipofectamine mixture was used for cotransfection of HEK-293T cells (4 × 10^6^ cells per 10-cm dish), and the cells were incubated at 37°C in a 5% CO_2_ incubator. After overnight incubation for 16 h, the transfected cells were replenished with fresh medium for subculture. At 48 h posttransfection, the pseudovirus-containing culture medium was collected by centrifugation at 1,000 × *g* for 10 min to remove unwanted cells or large debris, followed by passing the clarified medium through a 0.45-μm-pore filter (Millipore Corporation. Billerica, MA, USA). The virus can be stored at 4°C for immediate use or frozen at –80°C. Pseudovirus titers were determined using the p24 ELISA kit. (TaKaRa Bio).

### RBD-specific IgG ELISA.

To quantitatively measure IgG antibodies targeted to SARS-CoV-2 RBD, an indirect ELISA using anti-SARS-CoV-2 IgG1 monoclonal antibody (CR3022) as a quantitative standard was used (30–33). Ninety-six-well ELISA plates (Thermo Fisher) were coated for 15 h at 4°C with purified SARS-CoV-2 Alpha variant/B.1.1.7 RBD (Genetex, catalog no. GTX136014-pro) or Delta variant/B.1.617.2 RBD (GeneTex, catalog no. GTX136332-pro) diluted in carbonate-bicarbonate buffer to a concentration of 1 μg/mL. The plates were washed with 0.05% phosphate-buffered saline plus Tween 20 (PBST), followed by adding PBS containing 1% bovine serum albumin (BSA) and blocking at room temperature for 1 h, and then the plates were washed. Serum samples were diluted to 1:50 with a dilution buffer consisting of 1% BSA. A six-point standard curve was established using CR3022-IgG1 starting at 2 mg/mL and using 2-fold serial dilutions with dilution buffer. Sample serum and standards were added to corresponding wells and incubated for 1 h at room temperature, followed by washing. Target IgG antibodies were detected with anti-human IgG conjugated with horseradish peroxidase (HRP) (1:90,000). This detection antibody was added to the plate and incubated for 30 min at room temperature. After washing, TMB (3,3′,5,5′-tetramethylbenzidine) substrate (Invitrogen) was added to each well, the mixture was incubated for 5 min, and the reaction was stopped with 1 M H_2_SO_4_. The optical density was measured at 450 nm (OD_450_) with subtraction of the OD_570_ as a reference wavelength on an ELISA reader (BioTek). Anti-RBD antibody titers were calculated by interpolating onto a standard curve and correcting for sample dilution; 1 U/mL was defined as the equivalent reactivity seen with 1 mg/mL CR3022, and the cutoff value was defined as the mean OD_450_ value of prepandemic sera + 3 standard deviations (SD). All experiments were performed in duplicates.

### Neutralization assay with pseudotyped SARS-CoV-2 (pVNT_50_).

BHK-21/ACE2 cells were seeded at 4 × 10^4^ in a 24-well plate 16 h before the experiment. For the neutralization assay, 40 μL of heat-inactivated serum was started with a 1:16 dilution in complete medium containing 2% FBS, followed by 2-fold serial dilutions in duplicate samples and then incubation with 40 μL of pseudovirus (1 ng p24) for 1 h at 37°C. On the day of infection, the cells were washed twice with PBS, 100 μL of serum-virus mixture was added to the cells, and the cells were incubated for 48 h. The cells were quenched by adding 100 μL of BrightGlow luciferase substrate (Promega) directly to each well, and the luciferase activity was measured with Synergy H4 luminometer (BioTek). Background values, monitored from uninfected cells, were consistently below 400 relative luminescence units (RLU), and prepandemic sera were used to set as the negative control for the neutralization assay, with sera started at a dilution of 1:16, giving results in the range of the background RLU levels. A pVNT_50_ of >1:16 serum dilution was regarded as positive.

### Neutralization assay with infectious SARS-CoV-2 (PRNT_50_).

Serum samples were heated at 56°C for 30 min to inactivate complement; 2-fold serial dilutions, starting at a concentration of 1:5, were then mixed with an equal volume of viral solution containing 100 PFU of SARS-CoV-2. The serum-virus mixture was incubated for 1 h at 37°C in a humidified atmosphere with 5% CO_2_. After incubation, the mixture at each dilution was added to Vero E6 cells and the cells were incubated at 37°C for 1 h. Cells were subsequently cultured with DMEM containing 2% FBS and 1.4% methylcellulose for 72 h. After culturing, plaques were stained and counted. Neutralizing antibody titers were defined as the reciprocal of the maximum dilution of serum that reduced the virus titer by 50% compared to the negative-control sera, and PRNT_50_ values below 1:5 serum dilution were considered negative.

### Isolation of PBMCs.

Peripheral blood mononuclear cells (PBMCs) derived from 29 participants were isolated from anticoagulant-treated whole blood using Ficoll-Paque PLUS density gradient medium (Cytiva no. 17144003). To isolate PBMCs, blood diluted with PBS was gently layered over an equal volume of Ficoll in a Falcon tube and centrifuged for 30 min at 400 × *g* without braking. Four layers formed, each containing different cell types. The second layer contained PBMCs. These cells were removed by using a Pasteur pipette and added to warm medium or PBS to wash away any remaining platelets. The pelleted cells were then counted, and the percentage of viability was estimated using trypan blue staining. Isolated PBMCs were stored in liquid nitrogen until used in the ELISpot assay.

### ELISpot assay.

The amount of antigen-specific IFN-γ- or IL-2-secreting T cells was evaluated by ELISpot assays. Cryopreserved PBMCs were rapidly thawed and allowed to rest overnight. Cells were dispensed at 1 × 10^5^ cells per well for the IFN-γ or IL-2 ELISpot assay (human IFN-γ or IL-2 ELISpot kit; R&D Systems). The cells were stimulated with a pool of peptides consisting mainly of 15-mer sequences with 11-amino-acid overlap, covering the immunodominant sequence domains of the S protein of the SARS-CoV-2 wild type (PepTivator SARS-CoV-2 Prot_S; Miltenyi Biotec) or Delta variant (PepTivator SARS-CoV-2 Prot_S B.1.617.2; Miltenyi Biotec), and incubated at 37°C for 24 h. Cells stimulated with PHA-M (phytohemagglutinin, M form) (Thermo Fisher) served as the positive control, and all participants had more than 120 spot-forming cells (SFCs)/10^5^ PBMCs for both IFN-γ and IL-2. IFN-γ or IL-2 release was detected following the instructions in the manual, and the spots were counted using an ELISpot reader (Cellular Technology, Ltd.). The mean SFC value of duplicate peptide pool stimulated PBMCs was calculated and normalized by subtracting the mean of the negative-control replicates (control medium), and the cutoff value for background T cell responses was defined as the mean SFC value of seronegative PBMCs derived from healthy unvaccinated donors + 3 SD (9.5 SFCs/10^6^ PBMCs). The results were expressed as SFC per million PBMCs.

### Ethics.

This study was approved by the Tri-Service General Hospital (TSGHIRB no. B202105056). Informed consent was obtained from all enrolled participants. Work with infectious SARS-CoV-2 was approved by the Institutional Biosafety Committee (IBC) and was performed in the high-biocontainment biosafety level 3 (BSL-3) facilities of the Institute of Preventive Medicine (IPM), National Defense Medical Center (NDMC), which are approved for such use by the Taiwan Centers for Disease Control, under license no. D1-109-0030#1123 and D1-111-0017#2028 for institutional guidelines.

### Statistical analysis.

Statistical analyses were performed using GraphPad Prism 5. Anti-RBD IgG titers and pVNT_50_ and PRNT_50_ values were described as medians and IQRs. A nonlinear sigmoidal 4PL model was used to determine the pVNT_50_ and PRNT_50_ for each serum sample. The measured statistical significance for pseudovirus or infectious virus neutralization assays (pVNT_50_ and PRNT_50_) was calculated among experiments by one-way analysis of variance (ANOVA) with Tukey’s multiple-comparison test. Statistical significance of pVNT_50_ for each of the vaccination schedules against SARS-CoV-2 variants was calculated by two-tailed Friedman test with Dunn’s multiple-comparison test. Simple linear regression and Pearson correlation analysis were conducted to determine the correlation coefficients between anti-RBD IgG titers and pVNT_50_ or PRNT_50_ values. A two-tailed Student's *t* test was performed on the anti-RBD IgG titers and neutralizing titers between the different variants.
