# Supplementary material for: Boosting with Multiple Doses of mRNA Vaccine after Priming with Two Doses of Protein Subunit Vaccine MVC-COV1901 Elicited Robust Humoral and Cellular Immune Responses against Emerging SARS-CoV-2 Variants
Source: Microbiol Spectr. 2022 Aug 25;10(5):e00609-22. doi: 10.1128/spectrum.00609-22 (PMC9602831; doi:10.1128/spectrum.00609-22)
Supplement: Supplemental file 1 — Supplemental material. Download spectrum.00609-22-s0001.pdf, PDF file, 0.3 MB [file spectrum.00609-22-s0001.pdf]

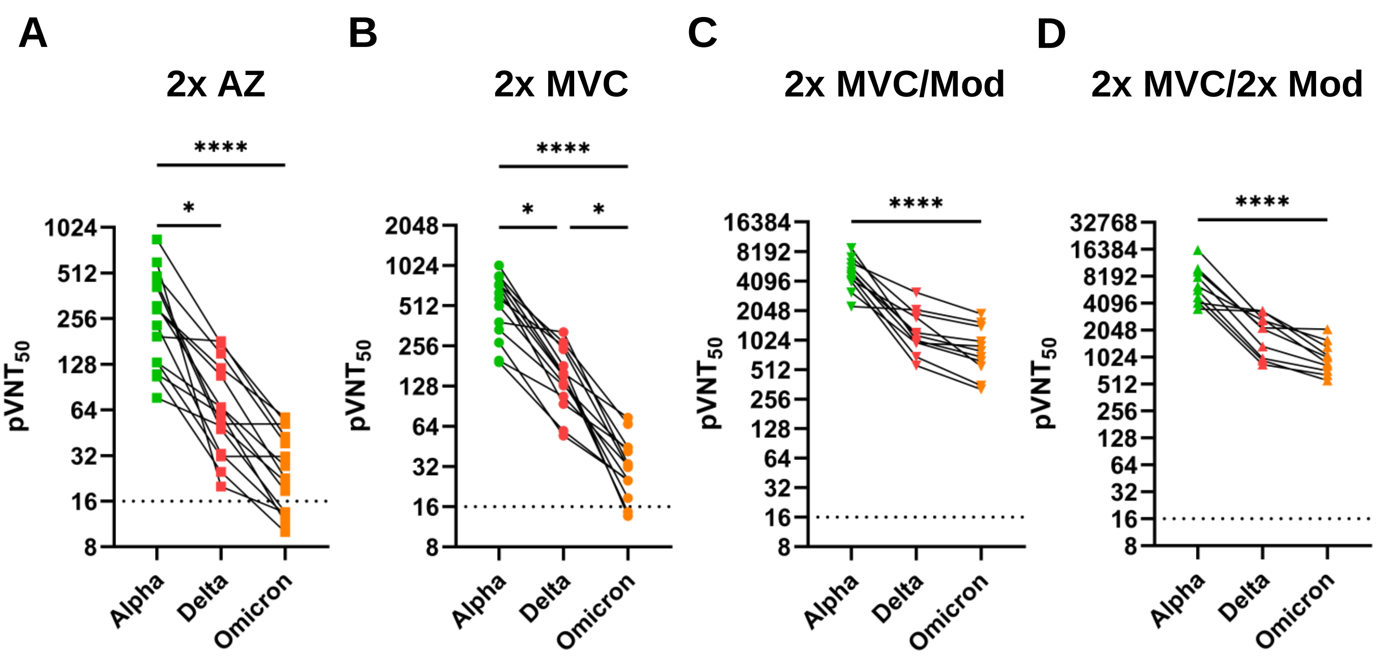

Supplementary Fig. S1. Comparison of the differences between alpha, delta and omicron variants of pseudovirus neutralization ($pVNT_{50}$). (A) 2x AZ, (B) 2x MVC, (C) 2x MVC/Mod and (D) 2x MVC/2x Mod. Duplicates were performed for each tested sample. Measured statistical significance was calculated among SARS-CoV-2 variants by two-tailed Friedman test with Dunn's multiple comparison. The dotted line represents the cut-off value for each assay. Asterisks indicate statistical significance, *$p<0.05$, ****$p<0.0001$.

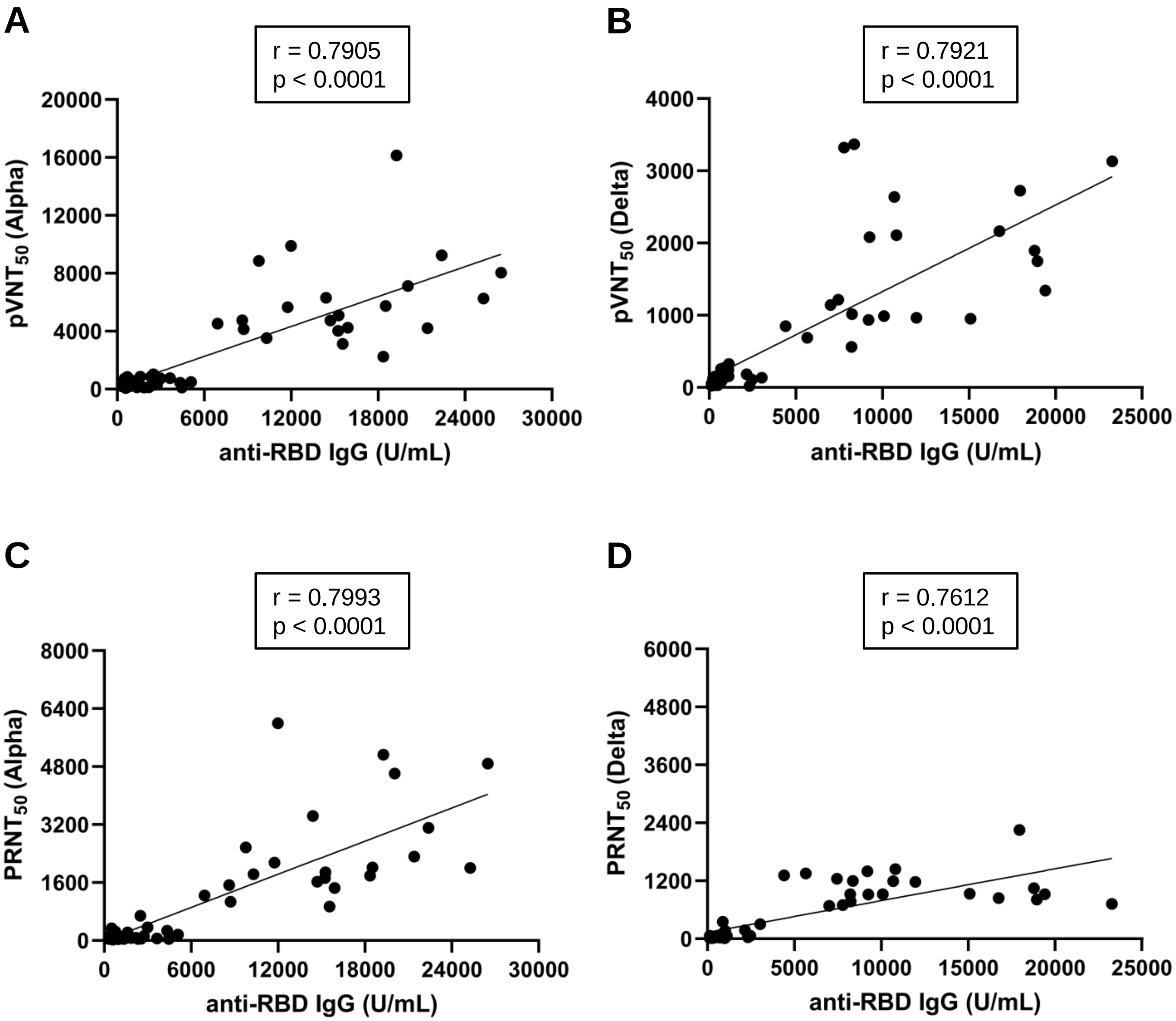

Supplementary Fig. S2. Correlation between anti-RBD IgG titers and neutralization titres ($pVNT_{50}$ or $PRNT_{50}$). (A) anti-RBD IgG titers (alpha variant) vs $pVNT_{50}$ (alpha variant), (B) anti-RBD IgG titers (delta variant) vs $pVNT_{50}$ (delta variant), (C) anti-RBD IgG titers (alpha variant) vs $PRNT_{50}$ (alpha variant), (D) anti-RBD IgG titers (delta variant) vs $PRNT_{50}$ (delta variant). Simple linear regression and Pearson correlation analysis were conducted to determine the correlation coefficients.

Supplementary Table 1- Neutralization potency of each participant against pseudovirus and infectious virus

Gender	Age	Underlying disease	Standard prime doses	Booster doses	$pVNT_{50}$ ($2^{nd}/3^{rd}/4^{th}$ dose)			$PRNT_{50}$ ($2^{nd}/3^{rd}/4^{th}$ dose)	
					Alpha variant	Delta variant	Omicron variant	Alpha variant	Delta variant
M	61	Hypertension	2x AZ		77	50	22	25	10
F	44		2x AZ		294	107	23	69	23
M	40		2x AZ		414	48	14	122	37
M	28		2x AZ		194	181	43	49	18
M	40		2x AZ		110	60	12	42	14
F	39		2x AZ		603	20	13	227	63
F	32		2x AZ		132	67	27	44	14
M	42		2x AZ		231	33	13	58	19
M	23		2x AZ		440	52	52	153	59
M	23		2x AZ		488	150	39	161	62
F	23		2x AZ		310	67	19	92	34
M	41		2x AZ		295	121	57	78	32
F	32		2x AZ		852	175	53	272	178
F	50		2x AZ		106	25	10	37	13
F	45	Hypertension	2x AZ		132	32	32	46	17
M	25		2x MVC	2x Mod	578/4757/5742	256/1215/990	45/990/721	133/1882/2014	160/934/923
F	29		2x MVC	2x Mod	740/5666/8046	273/1142/1343	67/789/824	284/2152/3436	301/922/924
M	62	Hypertension	2x MVC	2x Mod	194/2249/3522	59/2082/3321	25/1548/1328	43/935/1069	29/1244/1441
M	55	Hypertension	2x MVC	2x Mod	199/3126/4134	107/953/3370	33/889/1070	59/1244/1450	32/772/2254

F	32		2x MVC	2x Mod	726/5096/6305	94/966/2639	44/623/1579	225/2004/3113	31/814/1312
M	51		2x MVC	2x Mod	751/6250/9244	154/3133/2167	15/1896/2096	253/2317/4885	45/1353/1196
M	43		2x MVC	2x Mod	1024/8855/16143	181/1016/2725	14/690/875	681/4612/5994	57/915/1398
F	56	Hyperlipidemia	2x MVC	2x Mod	511/4203/4236	135/1749/848	26/552/644	131/1726/1624	39/1048/698
M	54		2x MVC	2x Mod	639/4523/4738	152/690/936	19/351/563	201/1793/1828	41/724/841
F	51		2x MVC	2x Mod	841/7130/9897	160/1896/2109	74/1409/1024	337/2569/5129	56/1177/1193
F	45		2x MVC	1x Mod	386/4029	324/562	34/322	118/1528	351/684
F	34		2x MVC		339	130	42	76	35
F	32		2x MVC		852	242	32	363	102
F	44		2x MVC		271	55	26	69	22
